# Hepatitis E

**DOI:** 10.1186/1471-2334-14-S2-S20

**Published:** 2014-05-23

**Authors:** Harry Dalton

**Affiliations:** 1 Royal Cornwall Hospital, Truro, TR1 3LJ, UK

## 

Until recently, HEV was thought not to occur in developed countries such as the UK, except in travellers returning from endemic areas. However, it has now become clear that autochthonous (locally acquired) HEV is common in developed countries, and is considered an “emerging disease”. Autochthonous HEV has been documented in Europe, the USA, Canada, Japan, and New Zealand. HEV infection acquired in these areas differs from that in developing countries in a number of important aspects: it is caused by genotype 3 (and 4 in China and Japan); it most commonly affects middle-aged/elderly males; it is zoonotic with a porcine primary host. Although pig herds worldwide are infected with HEV genotype 3, and HEV has been detected in retail meats in the human food chain in a number of developed countries, the route of transmission is not fully understood since most cases of autochthonous infection are not obviously associated with pigs or pig products. HEV has also been transmitted by blood transfusion in a number of countries, and surprisingly high numbers of asymptomatic blood donors are viraemic at the time of donation: Germany 1:1200, Netherlands 1:2671, England 1:7000 and Sweden 1:7986.

Our understanding of the clinical range of HEV infection in humans has undergone a sea-change in recent years. Until 2008, HEV was thought to cause only acute self-limiting infection. However, it is now evident that HEV may cause persistent disease in immune-compromised solid organ transplant recipients, individuals with HIV and patients with haematological malignancy. Patients with chronic HEV infection have no symptoms, but some develop rapidly progressive liver cirrhosis.

The full clinical spectrum of HEV in developed countries is still emerging. In Cornwall (UK), we have been studying the disease prospectively for over a decade, one of the longest studies of its type in a developed country. HEV has important extra-hepatic manifestations, which deserve further investigation. For example, we have shown that HEV is neuropathogenic, and can cause a wide range of neurological illness. In particular, very recent data suggests that Guillain-Barré syndrome and neuralgic amyotrophy are associated with locally acquired HEV in approximately 5% and 10% of cases respectively.

